# Sleep Disturbance Affects Immune Factors in Clinical Liver Cancer Patients

**DOI:** 10.3390/curroncol29100628

**Published:** 2022-10-20

**Authors:** Zuoyun Wang, Yan Wang, Jing Huang, Jietian Xu, Fangzhen Chen, Zidan Zhu, Lingling Gao, Jie Qin, Binbin Liu, Chunmin Liang

**Affiliations:** 1 Laboratory of Tumor Immunology, Department of Human Anatomy and Histoembryology, School of Basic Medical Sciences and Jing’an District Central Hospital of Shanghai, Shanghai Medical College, Fudan University, Shanghai 200030, China; 2 Liver Cancer Institute, Zhongshan Hospital, Fudan University, Shanghai 200030, China; 3 Key Laboratory of Carcinogenesis and Cancer Invasion, Ministry of Education, Shanghai 200030, China; 4 Department of Anethesiology, Cancer Hospital of Fudan University, Shanghai 200030, China

**Keywords:** liver cancer, sleep disturbances, PSQI, CD3^+^ T cells, NK cells

## Abstract

Background: Sleep–wake disturbance is prevalent in patients with liver cancer, but there is no direct evidence of its association and related biological mechanisms. Our study was to assess quality of sleep and to describe prevalence of sleep disturbances in patients with different etiologies of liver cancer, especially to explore whether sleep quality influences immune factors. Methods: A total of 210 patients with liver cancer from August 2015 to December 2015 were randomly divided into two groups including HBV cirrhosis and non-HBV cirrhosis. The Pittsburgh Sleep Quality Index (PSQI) was used to evaluate their sleep quality, and then 202 patients enrolled in this study were divided into two groups according to their PSQI scores: PSQI ≤ 5 and PSQI > 5. The association between sleep disturbances and immune factors was analyzed by logistic regression models. Results: A total of 56.9% of liver cancer patients experienced poor sleep quality (PSQI > 5). The prevalence of sleep disturbances was significantly higher in patients with liver cancer of non-hepatitis B virus (HBV) cirrhosis than with that evolving from HBV cirrhosis (66.7% vs. 50%, *p* = 0.018). In non-HBV cirrhosis liver cancer patients, the PSQI > 5 group had a higher percentage of CD3^+^ T cells (71.06 ± 11.07 vs. 63.96 ± 14.18, *p* = 0.014) and lower natural killer (NK) cells (14.67 ± 9.65 vs. 20.5 ± 10.77, *p* = 0.014) compared with patients with PSQI ≤ 5. Logistic regression further confirmed that liver cancer patients without HBV cirrhosis are more prone to experience poor sleep with increased CD3^+^ T cells (OR = 1.07, 95% CI = 1.01–1.13, *p* = 0.030) and decreased NK cells (OR = 0.92, 95% CI = 0.85–0.98, *p* = 0.014). Our results indicate that increased CD3^+^ T cells and decreased NK cells are both associated with sleep disturbances in patients with liver cancer of non-HBV cirrhosis. Conclusions: Most liver cancer patients suffer from sleep disturbances, especially evolving from non-HBV cirrhosis. A rise in CD3^+^ T cells and a reduction in NK cells are associated with sleep disturbances in patients with liver cancer of non-HBV cirrhosis.

## 1. Introduction

Sleep is very important to human health and we usually spend one third of our life sleeping [1]. Cumulative evidence indicates that sleep disturbance significantly increases the risk of inflammatory diseases, such as cardiovascular disease, diabetes, depression, cancer and all-cause mortality [2]. However, with the development of modern society, the shift to an urban lifestyle has caused a decline in population sleep duration and sleep quality over the past few decades [3,4]. Especially in cancer patients, the risk of developing sleep disturbances was approximately three times higher than in the general population [5,6], with an estimated prevalence from 31% to 59% [7,8]. The high incidence rate of sleep disorders in cancer patients is often associated with poor health, and the potential biological mechanisms contributing to sleep problems need further research and exploration.

Previous studies support that those predisposing factors of cancer-associated sleep disturbances are multifactorial, including stress associated with the diagnosis and treatment of cancer (such as corticosteroids, chemotherapy, sedative hypnotics and monoclonal antibodies) [9]. At this point, related works explain one potential mechanism involving dysfunction of the immune system [2]. Moreover, sleep disturbances cause the alteration of numbers and function of immune cells (including CD4^+^ T cells, B cells, NK cells and neutrophils) and a significant increase in the production of pro-inflammatory cytokines via neuroendocrine and circadian regulation [2]. It was found that 72 h paradoxical sleep deprivation (PSD) reduced the number of neutrophils and monocytes in bone marrow, but induced an accumulation of neutrophils and monocytes in peripheral blood [10]. In turn, infection and administration of pro-inflammatory cytokines also cause alteration to sleep modes, especially slow wave sleep (SWS) [11]. Moreover, chronic sleep restriction can accelerate carcinogenesis and tumor growth in a mouse model mediated by immunological mechanisms, such as tumor-associated macrophages and toll-like receptor 4 (TLR4) signaling [12,13]. Therefore, we speculate that the immune system might be involved in cancer-related sleep disturbances, and it is of great significance to study this relationship in clinical patients. 

Liver cancer is one of the most common malignant tumors at present, and its death rate ranks third in the world, which is the fourth leading cause of cancer death worldwide, with about 70% of liver cancer occurring in East and South-East Asia. We chose Zhongshan Hospital of Fudan University in Shanghai to collect liver cancer cases for our study, most of which are from the Chinese population. Numerous studies have now described sleep disturbances in different types of cancer patients, but mostly focused on liver cancer [14]. Understanding the prevalence of sleep disturbances in patients with different etiologies for liver cancer and its biological mechanisms is necessary on account of the high incidence and mortality of liver cancer. It is necessary to study the relationship between sleep disturbances and liver cancer. Specifically, in China alone the total number of cases and deaths accounts for about 50% [15]. Meanwhile, etiologies of liver cancer mainly include HBV infection, hepatitis C virus (HCV) infection, alcohol consumption, nonalcoholic steatohepatitis and other causes. Among them, HBV infection is the most common cause of liver cancer [16]. The purposes of our study were to evaluate the duration and quality of sleep by using self-reported questionnaires, and to describe and compare the prevalence of sleep disturbances in diverse etiologies of liver cancer patients (including evolving from HBV liver cirrhosis and non-HBV cirrhosis). Our other goal is to reveal the mediating role of immune surveillance in sleep disturbances. It is of great significance to provide clinical guidance to clinicians for supervision and treatment of liver cancer patients via improving sleep quality.

## 2. Materials and Methods

### 2.1. Participants

A total of 210 patients with liver cancer were enrolled in this study from Liver Cancer Research Institute at Zhongshan Hospital of Fudan University in Shanghai, China, from August to December 2015. They were hospitalized for treatment in liver internal medicine. Patients received transcatheter arterial embolization (TAE). These patients almost all belong to the Asian Chinese population and the number of samples has certain finitude. The inclusion criteria for the study were as follows: (1) Biopsy proven diagnosis of liver cancer: primary hepatocellular carcinoma, intrahepatic cholangiocarcinoma, colorectal cancer liver metastasis, neuroendocrine carcinoma of the liver, and primary lung or bone cancer with liver metastasis; (2) Age: at the age of 21 years or older; (3) The ability to accomplish questionnaires. All participants have given written informed consent before the data collection. In this study, we divided the participants into two groups according to etiology: evolved from HBV cirrhosis (*n* = 120) and non-HBV cirrhosis (*n* = 90).

### 2.2. Measurements

#### Demographic, Disease Associated Variables and Immune Factors

Demographic variables included the patients’ gender and age, which were obtained from the information filled out on the PSQI questionnaire (Supplementary File S1). Next, 118 cases of HBV cirrhosis and 84 cases of non-HBV were taken into account for the following analysis. Clinical data and immune-related parameters were retrospectively collected from the hospital’s electronic medical record database. The former clinical information of the patients included presence or absence of HBV cirrhosis, diabetes and hypertension, levels of C-reactive protein (CRP), alpha-fetoprotein (AFP), carcinoembryonic antigen (CEA) and carbohydrate antigen 19-9 (CA19-9). The latter information contained blood routine examination (numbers and percentages of white blood cell (WBC), lymphocyte (LY), neutrophils (NEUT) and monocytes (MONO), and cellular immunity examinations (percentages of B cells, CD3^+^, CD4^+^ and CD8^+^ T cells and NK cells, ratio of CD4^+^/CD8^+^ T cells) were performed by flow cytometry.

### 2.3. Sleep Disturbance

PSQI has become a popular instrument which is widely used in cancer research to assess sleep quality. Sleep disturbance is measured by the PSQI, which is a 19-item self-reported questionnaire assessing the sleep quality of the study populations during the last month. The 19 items comprise seven component scores: subjective sleep quality, sleep latency, sleep duration, habitual sleep efficiency, sleep disturbances, use of medication, and daytime dysfunction. A sum of all seven component scores is the PSQI global score ranging from 0 to 21. PSQI > 5 represents poor sleep quality, namely sleep disturbances, while PSQI ≤ 5 indicates good sleep [17]. 

### 2.4. Statistical Analysis

Characteristics are presented as percentages or mean ± SD. The Chi-square test was used for comparison of categorical variables according to etiology of liver cancer or sleep quality groups. Logistic regression models were then used to estimate odds ratios and 95% confidence intervals for the effects of sleep variables on the risks of frailty. The analyses were adjusted for potential confounding factors, including age, gender, hypertension, diabetes and inflammatory biomarkers. A two-tail *p* value of less than 0.05 was considered statistically significant. Statistical analyses were performed with IBM SPSS 22.

## 3. Results

### 3.1. Demographic and Immune Factors for Study Populations

Initially, 210 patients with liver cancer participated in this study, but in the end, only 202 patients completed the PSQI questionnaire verification. Among these 202 patients, 58.4% (*n* = 118) of participants were evolved from HBV cirrhosis, an additional 41.6% (*n* = 84) were other etiological factors, namely non-HBV cirrhosis. We divided all patients into two groups according to these different etiologies. Demographic characteristics of these clinical patients are shown in Table 1. The mean age was 59.1 years and the majority (82.2%) were males. There were no significant differences in sex, age, diabetes and hypertension between patients with liver cancer evolving from HBV cirrhosis and non-HBV cirrhosis (Table 1).

**Figure 1:**
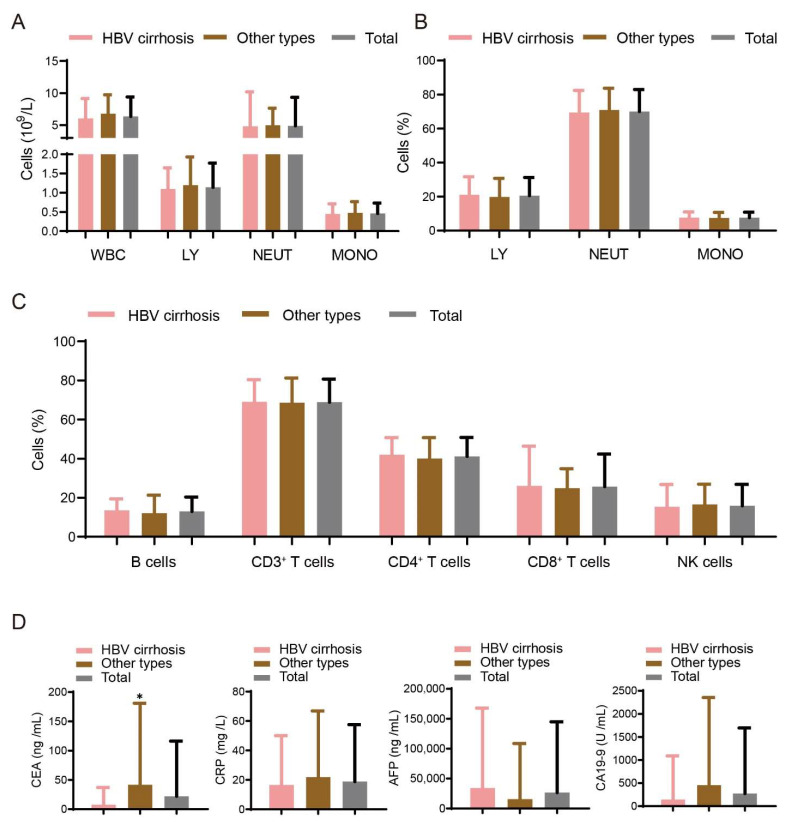
Disease-associated variables in two etiologies of patients with liver cancer. (**A**–**C**) Numbers and percentages of immune cells are detected in HBV cirrhosis (*n* = 118) patients and other types (*n* = 84) patients. (**D**) The concentrations of CRP and liver cancer biomarkers (AFP, CEA and CA19-9) were also detected in the blood of the two groups of patients. Data are shown as means ± SD. * *p* < 0.05.

Numbers and percentages of immune cells, concentrations of CRP and liver cancer biomarkers (AFP, CEA and CA19-9) were detected in the blood of the two groups of patients. There were no significant differences in WBC (white blood cell); LY (lymphocyte); MONO (monocyte); NEUT (neutrophil) and NK (natural killer) cells in the patients with liver cancer evolving from HBV cirrhosis and non-HBV cirrhosis (Figure 1A–C). Meanwhile, we found that the CEA level in blood drawing was significantly higher (41.94 ± 139.34 vs. 7.83 ± 29.23, *p* = 0.013) in patients with liver cancer of HBV cirrhosis than those of non-HBV cirrhosis (Figure 1D). 

### 3.2. Prevalence of Sleep Disturbances in Patients with Liver Cancer of Different Etiologies

The sleep quality of patients was presented in Table 2. The mean PSQI score of the entire group of participants was 6.91 ± 3.73. A total of 56.9% of the patients reported sleep disturbances (PSQI > 5). Interestingly, there were significant differences in sleep quality (*p* = 0.043) and prevalence of sleep disturbances (*p* = 0.018) between liver cancer patients with hepatitis B cirrhosis and those without hepatitis B cirrhosis. Compared to HBV cirrhosis patients (6.46 ± 3.79), patients of non-HBV cirrhosis (7.54 ± 3.58) had poorer sleep quality. Additionally, the prevalence of sleep disturbances was higher in patients without HBV cirrhosis (66.7%) than HBV cirrhosis (50%).

### 3.3. Association between Sleep Disturbances and Immune Factors 

**Figure 2:**
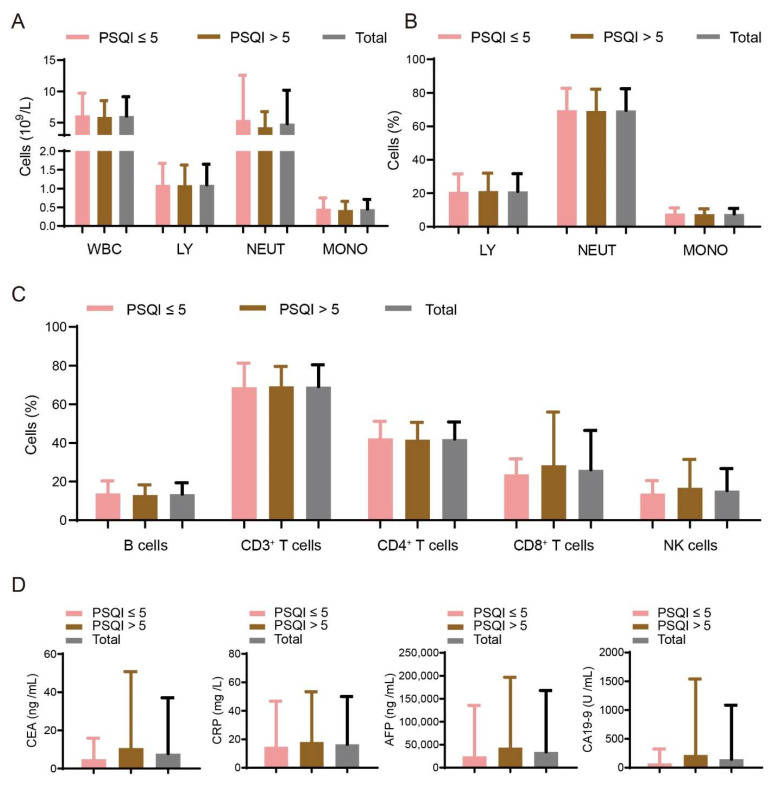
Association of sleep disturbances with demographic and immune variables in the group of HBV cirrhosis. (**A**–**C**) Numbers and percentages of immune cells are detected in PSQI ≤ 5 (*n* = 59) patients and PSQI > 5 (*n* = 59) patients with HBV cirrhosis. (**D**) The concentrations of CRP and liver cancer biomarkers (AFP, CEA and CA19-9) were also detected in the blood of the two groups of patients according to PSQI scores (PSQI ≤ 5 patients and PSQI > 5 patients).

We further divided the two groups with or without HBV cirrhosis into PSQI ≤ 5 and PSQI > 5 according to their PSQI scores. In the group with HBV cirrhosis, the numbers and percentages of immune cells from blood drawing did not show significant differences between the PSQI ≤ 5 and PSQI > 5 groups (Figure 2A–C). The concentrations of CRP and liver cancer biomarkers (AFP, CEA and CA19-9) also detected no difference (Figure 2D). The results suggest that the hepatitis B virus had a greater impact on liver cancer progression than the sleep disturbances.

**Figure 3:**
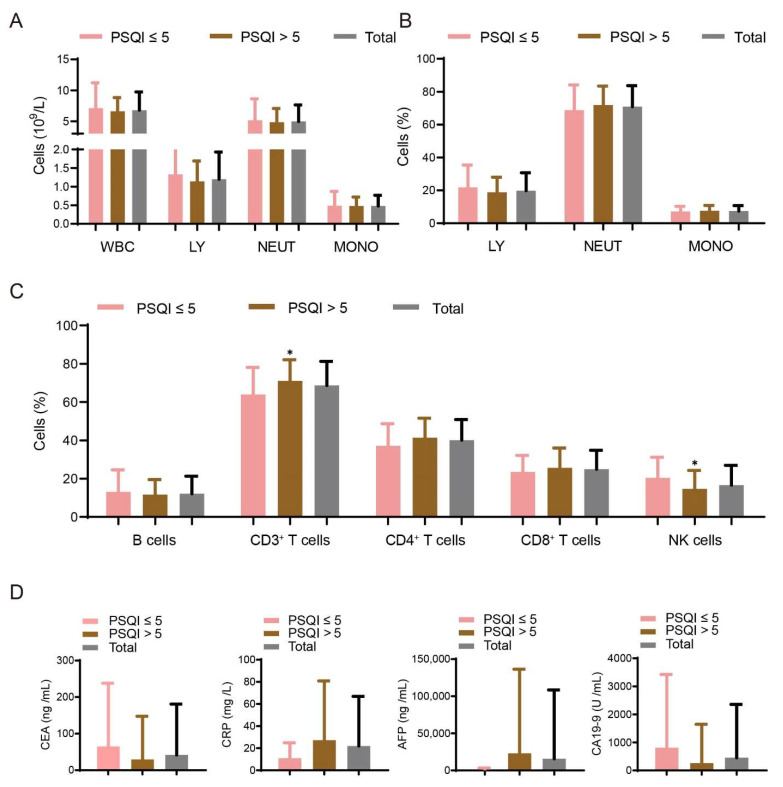
Association of sleep disturbances with demographic and immune variables in patients with non-HBV cirrhosis. (**A**–**C**) Numbers and percentages of immune cells are detected in PSQI ≤ 5 (*n* = 28) patients and PSQI > 5 (*n* = 56) patients without HBV cirrhosis. (**D**) The concentrations of CRP and liver cancer biomarkers (AFP, CEA and CA19-9) were also detected in the blood of the two groups of patients according to PSQI scores (PSQI ≤ 5 patients and PSQI > 5 patients). Data are shown as means ± SD. * *p* < 0.05.

We also detected the numbers and percentages of immune cells from blood drawing in the group without HBV cirrhosis (Figure 3A,B). However, in the group without HBV cirrhosis, patients with PSQI > 5 had a higher percentage (71.06 ± 11.07 vs. 63.96 ± 14.18, *p* = 0.014) of CD3^+^ T cells in comparison with patients with PSQI ≤ 5 (Figure 3C). However, the percentage of NK cells was lower in patients with PSQI > 5 (14.67 ± 9.65) than the other group (20.5 ± 10.77, *p* = 0.014) (Figure 3C). The concentrations of CRP and liver cancer biomarkers (AFP, CEA and CA19-9) showed no difference (Figure 3D).

### 3.4. Logistic Regression Analyses between CD3^+^ T and NK Cells with Sleep Disturbances

**Figure 4:**
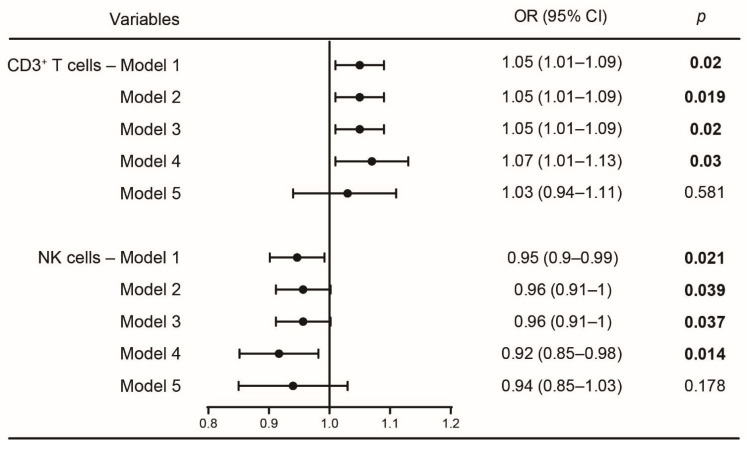
Logistic regression analysis of CD3^+^ T and NK cells associated with sleep disturbances in non-HBV cirrhosis patients. Model 1: without any adjustments; model 2: adjustment for age and sex; model 3: model 2 plus adjustment for diabetes and hypertension; model 4: model 3 plus adjustment for immune factors except CD3^+^ T and NK cells; Model 5: model 4 plus adjustment for CD3^+^ T or NK cells. PSQI ≤ 5 set as reference. OR, odds ratios; CI, confidence interval; NK, natural killer; PSQI, Pittsburgh sleep quality index. Bold values are statistically significant. Data are shown as means ± SD. *p* < 0.05.

To further confirm the association between immune cells and sleep disturbances, binary logistic regression analyses were used to demonstrate the effects of CD3^+^ T and NK cells in sleep disturbances of patients with liver cancer due to non-HBV cirrhosis. Figure 4 displayed the results of unadjusted and adjusted logistic regression models. Using PSQI ≤ 5 as a reference, the odds ratios (OR) of PSQI > 5 were presented in Figure 4. In unadjusted analyses, the percentages of CD3^+^ T cells and NK cells were significantly positively (OR_model1_ = 1.05, 95% CI = 1.01–1.09, *p* = 0.02) and negatively (OR_model1_ = 0.95, 95% CI = 0.9–0.99, *p* = 0.021) associated with an increased risk of sleep disturbances, respectively.

After adjustment for age and sex, the association between CD3^+^ T (OR_model2_ = 1.05, 95% CI = 1.01–1.09, *p* = 0.019) and NK cells (OR_model2_ = 0.96, 95% CI = 0.91–1, *p* = 0.039) with sleep disturbances remained. The significant correlation also existed after further adjustment for diabetes and hypertension variables in CD3^+^ T cells (OR_model3_ = 1.05, 95% CI = 1.01–1.09, *p* = 0.02) and NK cells (OR_model3_ = 0.96, 95% CI = 0.91–1, *p* = 0.037), respectively. Even after further adjustment for immune factors except CD3^+^ T cells and NK cells, the risk of poor sleep quality still increased by 7% with an elevation of CD3^+^ T cells percentages (95% CI = 1.01–1.13, *p* = 0.03). Furthermore, the risk of sleep disturbances increased by 8% with the decrease in NK cell percentages (95% CI = 0.85–0.98, *p* = 0.014). In summary, our results indicate that a rise in CD3^+^ T cells and a decrease in NK cells are both associated with sleep disturbances in patients with liver cancer of non-HBV cirrhosis.

## 4. Discussion

In the past, multiple studies have found that sleep quality is related to tumor progression, and our previous studies have shown that sleep disorders can impair immunity and promote tumor growth through immune cells in blood and peripheral immune organs in a mouse hepatocellular carcinoma model; however, there is a lack of research in clinical patients with liver cancer [18,19]. This study focuses on two types of patients with HBV cirrhosis and non-HBV cirrhosis. There are two emerging findings in this research. Firstly, a fairly large proportion (56.9%) of patients with liver cancer reported poor sleep; and the sleep quality of patients without hepatitis B cirrhosis was significantly poorer than those with hepatitis B cirrhosis. Secondly, in the subtype of patients with liver cancer of non-HBV cirrhosis, the elevation of CD3^+^ T cells and the reduction of NK cells were both associated with an increased risk of sleep disturbances even after adjustment for potential confounding factors (age, sex, diabetes and hypertension variables, immune cells except CD3^+^ T and NK cells). Our results suggest a novel treatment idea of improving sleep disturbances to relieve patients’ pain and get greater therapeutic effects via improving CD3^+^ T and NK cells.

Previous researches have reported that sleep disturbances are associated with many liver diseases, such as non-alcoholic fatty liver disease and liver cirrhosis [20]. Most of these liver diseases are closely related to liver cancer. The majority of previous studies in liver cancer patients focused on sleep disturbances increasing the risk of liver cancer [21,22] and sleep duration related to increased mortality in advanced cancer patients. While the samples which covered cancer patients of hepatobiliary–pancreatic system were heterogenous [22], our study populations were homogenous liver cancer patients. In addition, we collected and analyzed a fairly complete data of immune cells, and we also excluded potential confounding factors that may influence the association. Our study found that patients with liver cancer of non-HBV cirrhosis suffered from poorer sleep, and that dysfunction of CD3^+^ T and NK cells involves one of the mechanisms of cancer-associated sleep disturbances. This has implications for clinical workers paying more attention to supervising sleep disturbances in liver cancer patients. Recent analysis of treatment regimens for liver cancer has shown that, personalized drug dosage can improve the patients’ immune status and immune response to medical treatments thus increasing life quality and prognosis, including sleep quality [23,24]. This study also provided a significant correlation between immunity and sleep disorders in clinical HCC cases.

Indeed, this study has some limitations: (1) we collected clinical data and immune-related parameters and analyzed by logistic regression models, while a follow-up research of patients in different disease stages could improve the comprehensiveness of this study; (2) sleep quality was assessed by a subjective questionnaire, while there are more objective and sensitive methods for recording sleep, such as actigraphy and polysomnography; (3) the clinical samples scale should be larger and contain more information, such as collecting more samples with different races, and more information including tumor staging or liver function, to demonstrate the further reliable and objective results. However, we firstly and originally investigated sleep quality in different etiologies of patients with liver cancer, and explored immune factors as one of the mechanisms mediating cancer-associated sleep disturbances. Medicines that can promote sleep and improve organism’s immune function might be beneficial to tumor prevention, such as melatonin. It is indicated that men who report sleep problems have lower 6-sulfatoxymelatonin (6-STM, the primary melatonin metabolite) levels and are at increased risk for advanced or lethal prostate cancer [25]. Therefore, melatonin-associated medicines are expected to be applied for clinical liver cancer patients as a pharmacotherapy of sleep disturbances. In case of complex drug side effects for tumor patients, non-pharmacological sleep interventions as an adjunctive therapy, such as cognitive behavioral therapy and exercises which can significantly improve sleep quality, should be a safer choice.

## Figures and Tables

**Table 1:** Descriptive characteristics of patients with liver cancer evolving from HBV cirrhosis and non-HBV cirrhosis.

Characteristics	HBV Cirrhosis		Other Types		Total		*p*
	*n*	%	*n*	%	*n*	%	
Age, years							0.228
Mean	58.5		60		59.1		
SD	8.3		9.6		8.9		
Sex							0.258
Male	100	84.7	66	78.6	166	82.2	
Female	18	15.3	18	21.4	36	17.8	
Diabetes							0.200
Yes	9	7.6	11	13.1	20	9.9	
No	109	92.4	73	86.9	182	90.1	
Hypertension							0.188
Yes	23	19.5	23	27.4	46	22.8	
No	95	80.5	61	72.6	156	77.2	

HBV, hepatitis B virus; SD, standard deviation.

**Table 2:** PSQI score and prevalence of sleep disturbances in patients with liver cancer of HBV cirrhosis and non-HBV cirrhosis.

	HBV Cirrhosis	Others	Total	*p*
	*n* = 118	*n* = 84	*n* = 202	
PSQI score				
Mean	6.46	7.54	6.91	**0.043** ^a^
SD	3.79	3.58	3.73	
PSQI ≤ 5				
N (%)	59 (50)	28 (33.3)	87 (43.1)	**0.018** ^a^
PSQI > 5				
N (%)	59 (50)	56 (66.7)	115 (56.9)	

HBV, hepatitis B virus; PSQI, Pittsburgh sleep quality index; SD, standard deviation. Bold values are statistically significant. Data are shown as means ± SD. ^a^ *p* < 0.05.

## Data Availability

All data relevant to the study are included in the article.

## Supplementary Materials

The following supporting information can be downloaded at: https://www.mdpi.com/article/10.3390/curroncol29100628/s1, File S1: Pittsburgh Sleep Quality Index (PSQI).
