# Supplementary material for: Sleep Disturbance Affects Immune Factors in Clinical Liver Cancer Patients
Source: Curr Oncol. 2022 Oct 20;29(10):7943–52. doi: 10.3390/curroncol29100628 (PMC9600048; doi:10.3390/curroncol29100628)
Supplement: Supplementary file 1 [file curroncol-29-00628-s001.zip › curroncol-1923831-supplementary.pdf]

SUPPLEMENTARY

Supplementary File S1. Pittsburgh Sleep Quality Index (PSQI)

Name_______ Sex______ ID#________ Date_______ Age_____

Instructions:

The following questions relate to your usual sleep habits during the past month ONLY. Your answers should indicate the most accurate reply for the majority of days and nights in the past month. Please answer all questions.

1. During the past month, when have you usually gone to bed at night?

USUAL BED TIME______________________

2. During the past month, how long (in minutes) has it usually taken you to fall asleep each night?

NUMBER OF MINUTES___________________

3. During the past month, when have you usually gotten up in the morning?

USUAL GETTING UP TIME________________

4. During the past month, how many hours of actual sleep did you get at night? (This may be different than the number of hours you spend in bed.)

HOURS OF SLEEP PER NIGHT______________

For each of the remaining questions, check the one best response. Please answer all questions.

5. During the past month, how often have you had trouble sleeping because you…

(a) cannot get to sleep within 30 minutes

Not during the Less than Once or Three or more

past month______ once a week______ twice a week______ times a week_____

(b) Wake up in the middle of the night or early morning

Not during the Less than Once or Three or more

past month______ once a week______ twice a week______ times a week_____

(c) Have to get up to use the bathroom.

Not during the Less than Once or Three or more

past month______ once a week______ twice a week______ times a week_____

(d) Cannot breathe comfortably.

Not during the Less than Once or Three or more

past month______ once a week______ twice a week______ times a week_____

(e) Cough or snore loudly.

Not during the Less than Once or Three or more

past month______ once a week______ twice a week______ times a week_____

(f) Feel too cold.

Not during the Less than Once or Three or more

past month_____ once a week______ twice a week______ times a week_____

(g) Feel too hot.

Not during the Less than Once or Three or more

Past month_______ once a week______ twice a week______ times a week_____

(h) Had bad dreams.

Not during the Less than Once or Three or more

Past month_______ once a week______ twice a week______ times a week_____
(i) Have pain.
Not during the Less than Once or Three or more
Past month_______ once a week______ twice a week______ times a week_____
(j) Other reason(s), please describe________________________________
How often during the past month have you had trouble sleeping because of this?
Not during the Less than Once or Three or more
Past month_______ once a week______ twice a week______ times a week_____
6. During the past month, how would you rate your sleep quality overall?
Very good_______ Fairly good______ Fairly bad_______ Very bad _________
7. During the past month, how often have you taken medicine (Prescribed or "over the counter") to help you sleep?
Not during the Less than Once or Three or more
Past month_______ once a week______ twice a week______ times a week_____
8. During the past month, how often have you had trouble staying awake while driving, eating meals, or engaging in social activity?
Not during the Less than Once or Three or more
Past month_______ once a week______ twice a week______ times a week_____
9. During the past month, how much of a problem has it been for you to keep up enough enthusiasm to get things done?
No problem at all ________Only a very slight problem ________
Somewhat of a problem ________A very big problem ________
10. Do you have a bed partner or share a room?
No bed partner or do not share a room _____ Partner/ flatmate in other room ______
Partner in same room, but not same bed ________ Partner in same bed ________
11. If you have a bed partner or share a room, ask him/her how often in the past month you have had………
(a) Loud snoring.
Not during the Less than Once or Three or more
Past month_______ once a week______ twice a week______ times a week_____
(b) Long pauses between breaths while asleep.
Not during the Less than Once or Three or more
Past month_______ once a week______ twice a week______ times a week_____
(c) Legs twitching or jerking while you sleep.
Not during the Less than Once or Three or more
Past month_______ once a week______ twice a week______ times a week_____
(d) Episodes of disorientation or confusion during sleep.
Not during the Less than Once or Three or more
Past month_______ once a week______ twice a week______ times a week_____
(e) Other restlessness while you sleep: please describe______________________
Not during the Less than Once or Three or more
Past month_______ once a week______ twice a week______ times a week_____
